# Glycerine in Phthisis

**Published:** 1857-06

**Authors:** 


*Glycerine in Phthisis.*—Professor N. S. Davis in the Northwestern Medical and Surgical Journal, says on this subject: "For cases of tubercular disease in its early stage, before the cough is accompanied by much expectoration, we frequently prescribe—

℞. Glycerine, ℥ ij.
Iodide of Potassium, ʒ i.
Sulphate of morphine, grs. ij.

Mix, and give one teaspoonful before each meal and at bed time.

If the disease is farther advanced, and expectoration more copious, with rapidly increasing emaciation, we prefer the following—℞. Glycerine, ℥ ij; syrup of iodide of iron, ℥ ss; sulphate of morphine, grs. ij. Mix, and give one teaspoonful every four or six hours.

It is now two years since we commenced using the glycerine in the treatment of phthisis, generally combining it with some preparation of Iodine, and just enough morphine to allay cough and promote rest; and we have certainly derived more benefit from it than from any other one remedy.—*St. Louis Medical & Surgical Journal.*

